# New Thinking on Flame Retardants

**DOI:** 10.1289/ehp.116-a210

**Published:** 2008-05

**Authors:** Kellyn S. Betts

No one wants their bed, couch, chair, computer, or TV to catch on fire. “If an ordinary upholstered chair in your home gets ignited, it can essentially take your whole house down,” says Richard Gann, a senior research scientist at the U.S. National Institute of Standards and Technology’s (NIST) Building and Fire Research Laboratory. The most flammable part of a mattress or couch is its plastic polyurethane foam cushioning, he explains. Once a fire gets through a chair or mattress’s fabric covering and into this cushioning, it can start a catastrophic reaction that quickly leads to “flashover,” in which nearly everything combustible inside a room ignites simultaneously.

Until very recently, brominated flame retardants, especially polybrominated diphenyl ethers (PBDEs), were one of the main materials used to reduce the speed with which the plastic components of consumer goods including beds, couches, chairs, and electronics could be consumed by fire. However, growing evidence shows that PBDE compounds are escaping from the products they protect and making their way into the products’ users. Moreover, the chemicals may disrupt human thyroid hormone functioning and cause other health effects, prompting many nations to ban or suspend their use in new consumer goods. [For more information on the health effects of PBDEs, see “Unwelcome Guest: PBDEs in Indoor Dust, p. A202 this issue.]

Although bromine- and chlorine-containing flame retardants are still used in some products, the need for new alternatives is being driven by a confluence of policy, standards, and pressure from environmental groups. Europe banned the use of two formulations, PBDE pentaBDE and octaBDE, in 2004, the same year they were withdrawn from the North American market. A third compound, decaBDE, was banned 1 April 2008 by the European Court of Justice. Stateside, Maine has banned the use of decaBDE, the only PBDE still on the market in North America, in mattresses and residential upholstered furniture produced and sold in that state, and will extend the ban to electronics in 2010. Washington prohibits the use of decaBDE in mattresses and sets a process for a future ban in furniture and electronics if the state can identify a safer and feasible alternative that meets fire safety standards. Asian countries and other U.S. states have similar legislation in the works.

“Instead of adding new fire retardant chemicals that ultimately may be shown to cause health problems, we should be asking whether we need to use these chemicals or if there are other ways to achieve equivalent fire safety,” contends Arlene Blum, a biophysical chemist and visiting scholar at the University of California, Berkeley. “So many of the chemicals we have banned in the past were flame retardants—think about asbestos, polychlorinated biphenyls, polybrominated biphenyls, tris(2,3-dibromopropyl) phosphate, PBDEs—[and] they all ended up in the environment and in people,” she points out. “We need to think carefully about adding these sorts of chemicals to consumer products before there is adequate health information.”

## Policy Drivers

Two new standards from the U.S. Consumer Product Safety Commission (CPSC) are opening the door for innovative approaches for protecting consumer goods containing polyurethane foam from fire. The first took effect last year for mattresses. This standard is innovative in being the first in the United States to focus on the rate of heat release, which fire safety experts recognize is the main determinant of how quickly a fire can spread out of control to the flashover point, Gann says.

The mattress industry worked with NIST to develop the new standard test method to meet the CPSC regulation, which stipulates that no mattress may generate a peak heat release rate greater than 200 kilowatts when subjected to gas burners that mimic burning bedding. The CPSC estimates the new standard will prevent as many as 270 fire-related deaths and 1,330 injuries every year. Since this is a performance standard rather than a prescribed mattress design, it allows manufacturers to choose how to fabricate mattresses that comply with the regulation, Gann says.

One approach mattress manufacturers are using to meet the standard is to employ what is known in the industry as a barrier material, says Tom Ohlemiller, who was the project leader for the NIST team that developed the mattress test method. The barrier materials themselves may be inherently nonflammable, such as polyamides like Kevlar. Flammable barriers may be protected with proprietary fire retardant treatments such as decaBDE. However, Ohlemiller says the standard does not require such treatments for the polyurethane foam padding beneath the barrier, which some scientists believe is the source of some of the PBDE flame retardants that have escaped into people’s homes. Over the past year, scientists have reported detecting other flame retardants used in polyurethane foam in household dust.

The second new standard, which affects upholstered furniture, is still wending its way through the regulatory process. According to Nancy Nord, acting chairman of the CPSC, the new rule will address upholstered furniture fires without requiring the use of fire retardant chemicals. Under the new proposal, furniture manufacturers could meet the performance standard by using smolder-resistant cover fabrics or interior fire-resistant barriers to protect the furniture’s internal filling material. The standard was put out for public comment in the *Federal Register* on 4 March 2008 and is open for comment until May 19.

The furniture standard focuses on cigarettes as a source of fires because they are responsible for 90% of the fires involving upholstery, says Russell Batson, vice president of government affairs for the American Home Furnishings Alliance, an industry group. “You can get smolder resistance without relying on chemicals,” he says. However, Gann points out that “cigarette ignition resistance is going to be improved significantly anyway” due to the passage over the past four years of laws mandating that Canada and 24 U.S. states can sell only “fire-safe” cigarettes, which self-extinguish if left unattended. These laws affect nearly 60% of the North American population, according to the nonprofit Coalition for Fire-Safe Cigarettes.

Additionally, Alexander Morgan, a group leader at the University of Dayton Research Institute, says there is a lot of concern about barriers failing against ignition sources stronger than a cigarette, especially since smoking rates are declining in many developed nations, according to the World Health Organization. He says candles, hot electrical equipment, and short-circuiting laptops could easily penetrate these protective barriers.

This is a fundamental weakness of the barrier approach in light of several decades of fire safety data for furniture from the United Kingdom, which Morgan says has the world’s toughest flammability standards for polyurethane foam. “Yes, they do use flame retardants, but the level of fire safety of their products is very good and fire losses in the UK due to furniture fires are quite low or non-existent. When and if flashover occurs due to a furniture fire, the amount of pollution and carcinogens released from this one fire far overwhelms the production of potentially dangerous products from a flame retardant foam,” he says. Morgan argues that the solution may be to devise flame retardants that are less likely to escape from the materials that they protect, together with better product reclamation and recycling programs for flame retardant products so that the chemicals don’t end up into the environment.

Despite such concerns, Batson says the proposed standard is inspiring furniture manufacturers to investigate how barriers can be used to insulate the interior cushioning materials inside upholstered furniture. “Recent innovations in materials science, together with concerns about flame retardant toxicity and ecotoxicity have convinced people in the industry to try to design effective barrier materials for the market,” he says. The furniture industry is looking carefully at how mattress manufacturers construct fire-blocking barrier layers of fabric or “high-loft” materials such as batting rather than chemically loading the outer fabric layer, he says. “That approach and some of the technologies that are emerging in response to it is probably going to be useful in the furniture [industry], as well,” he says.

## Nanomaterials

One promising approach is to incorporate flame retardants into the materials themselves. A new company called G3 Technology Innovations (G3*i*) is pursuing that line of reasoning with its GreenShield FR™ treatment for polyester fabrics. Such fabrics are the basis of 90% of the products used in the contract textile industry—which produces all furniture, floor coverings, wall coverings, and window treatments used in commercial buildings and institutions—says Alex Qiao, G3*i* ’s co-founder and president.

The technology, which G3*i* co-founder and chief operating officer Suresh Sunderrajan and his business partners developed for different applications while previously employed at Eastman Kodak, revolves around the ability to attach different functional groups onto nanoparticles. “We are able to attach multiple sets of these [functional molecules] onto the particles,” he explains. For example, he says one set of the molecules might encompass the particles needed to allow the molecules to attach themselves to a fabric’s fibers, a second set might provide water and stain repellency, and a third set could involve flame retardancy. “All of this is built onto a [silica-based] backbone which is inherently nonflammable,” he explains. The GreenShield FR treatment “goes into the [polyester] fiber and becomes a permanent part of it,” Qiao says.

The company has also worked with a textile finisher called Preferred Finishing to develop new barrier materials that Qiao says can become integral parts of the fabric they protect because both are made of polyester resin. This confers an additional advantage of avoiding the use of melamine–formaldehyde resin, which is often used to bind other barriers to decorative fabrics, Sunderrajan points out. When the resin degrades, he explains, it releases formaldehyde, which the International Agency for Research on Cancer classifies as a known human carcinogen. The company says all of its technologies are based upon commercially available materials that have been tested individually for toxicity. Several furniture makers are now testing the G3*i* products.

Nanoclays are another material that could change the way consumer products are protected from combustion. Flame retardants made with naturally occurring clay called montmorillonite are poised to have a huge influence on future fire safety, Gann says. Scientists at NIST and Cornell University have been investigating how this clay can help reduce the amount of energy released during fires for more than a decade, says Jeffrey Gilman, a research chemist at NIST.

“When things burn, contrary to how it looks, it is not the solid that is burning. The solid breaks down to give you small fragments of molecules. These vaporize and mix with the air, and they burn there,” Gann explains. “The ‘nano-network’ formed by the nanoclays impedes this from happening,” he says. “If the [nanoclay] particles are appropriately spread out and dispersed through the host [material], they form sort of a gauze inside the material. It slows down significantly or even prevents the breakdown of material and the release of gas-phase combustible molecules,” he says.

The potential of nanoclays isn’t just theoretical. A company called Nanocor sells nanoclay-based flame retardants that are used in electronics, wires, cables, and decorative wallpapers, says Tie Lan, general manager for the company’s U.S. operations. “The fundamental nature of the nanoclay will make the material burn slower [and] lower the temperature of the flame,” he says, adding that the same clays are also used in nonclumping kitty litters.

Both Nanocor and Albemarle Corporation, one of the major flame retardant makers, sell flame retardants combining nanoclays with another major class of flame retardants based on metal hydroxides. The nanoclays synergistically improve how the metal hydroxide retardants perform, Gilman says. Combining the two flame retardants also improves how the plastics are processed, as well as their material properties. Nanoclays are appealing to plastics manufacturers because they can be added in relatively small amounts, on the order of a few percent by weight. This means both that they are unlikely to negatively affect the functionality of the plastic material to which they are added and that they are relatively inexpensive, Gann says.

More recently, the NIST researchers have also begun to look at other nanomaterials, including carbon nanotubes, layered hydroxides, and polyhedral oligomeric silsesquioxane nanocomposites that also contain silicon, says Gilman. Some nanomaterials, especially carbon nanofibers, appear to have promise for use in polyurethane foam, says Mauro Zammarano, a guest researcher from Italy evaluating these materials at NIST. Testing at NIST suggests carbon nanofibers are able to reduce the rate at which heat is released when polyurethane foam is burned.

However, Andrew Maynard, chief science advisor of the Project on Emerging Nanotechnologies, a nonprofit group associated with the Woodrow Wilson International Center for Scholars, cautions that the same properties that make the nanoparticles effective could also make them toxic. “With any sort of nanotechnology . . . [the] potential for harm is associated with the size and shape of the particles, as well as what they’re made of. That applies whether you’re looking at sunscreen, impregnated fabrics, or flame retardants,” he says. Scientists need to look carefully to determine if there is any way the nanomaterial-based flame retardants escape from the fabric or material in which they’re used and enter the environment, and whether people could be exposed to the nanoparticles, he says.

NIST has begun to work with the CPSC and Scripps Institution of Oceanography to evaluate whether any of these nanomaterial-based fire retardants are toxic, Gilman says. Dimitri Deheyn, a marine biologist at Scripps’ Marine Biology Division, is conducting some of this testing using brittle stars, which Deheyn says have nervous systems that function very similarly to mammals, including humans. He says the testing he has conducted to date suggests the surfactants used to ensure the nanomaterials disperse throughout the materials to which they are added may be more toxic than the nanomaterials themselves.

## Halogen-Free Electronics

The electronics industry is under pressure from environmental groups to remove potentially toxic compounds from their products, including the brominated flame retardants that were once widely used in electronics housings and cases and are still used extensively in printed circuit boards. At least nine leading electronics companies have pledged to remove brominated and/or halogenated flame retardants from some or all of their products, according to the Environmental Working Group.

The main way that companies are doing this is by using phosphorus-based flame retardants for casings and circuit boards, and using minerals such as nanoclays in combination with aluminum and magnesium hydroxide for the machinery’s wiring and cabling, says Morgan. However, he points out that companies and environmental watchdogs are scrutinizing some of these phosphorus-based retardants for potential health problems of their own; for example, some are suspected to be neurotoxicants when they break down in the environment, he points out. He says his experience testing how well different nonhalogenated flame retardants work suggests that reactive phosphorus-based retardants appear to be the best nonhalogenated flame retardants for printed circuit boards at this time, in terms of their effectiveness, long-term durability, sustainability, and environmental impact.

Trying to find halogen-free alternatives for electronic circuit boards involves significant trade-offs, stresses Fern Abrams, the director of government relations and environmental policy for IPC, an electronics industry association for manufacturers of printed circuit boards and other electronics components. She says the “holy grail” would be to develop materials for building and housing electronics that are inherently flame-resistant.

Morgan agrees. He says the aerospace industry currently uses some inherently non-flammable plastics, but they are too expensive for commodity-type applications such as electronics housings, given the industry’s profit margins. More recently, scientists have begun trying to develop plastic polymers that are inherently nontoxic and nonflammable.

One team involved in this effort is at the University of Massachusetts Amherst, where researchers have developed a new plastic polymer based on bishydroxydeoxybenzoin (BHDB) that releases water vapor rather than hazardous gases when it breaks down in a fire. “The great thing about BHDB is that . . . it is extremely fire-safe and does not contain halogenated additives,” says Bryan Coughlin of the university’s Polymer Science and Engineering Department, one of the new material’s co-inventors.

The Amherst researchers believe BHDB may prove to be cost-effective for use in some consumer products, including home furnishings and electronics. “We are currently trying to determine how well BHDB works in a variety of plastics formulations . . . including polyurethane foam,” says Todd Emrick, another co-inventor at the University of Massachusetts Amherst Polymer Science and Engineering Department. The fire safety experts at NIST say that they believe the material has a great deal of promise. But the biggest challenge, as Morgan points out, may be finding a company willing to make the investment needed to bring such an innovative technology to the marketplace.

## Figures and Tables

**Figure:**
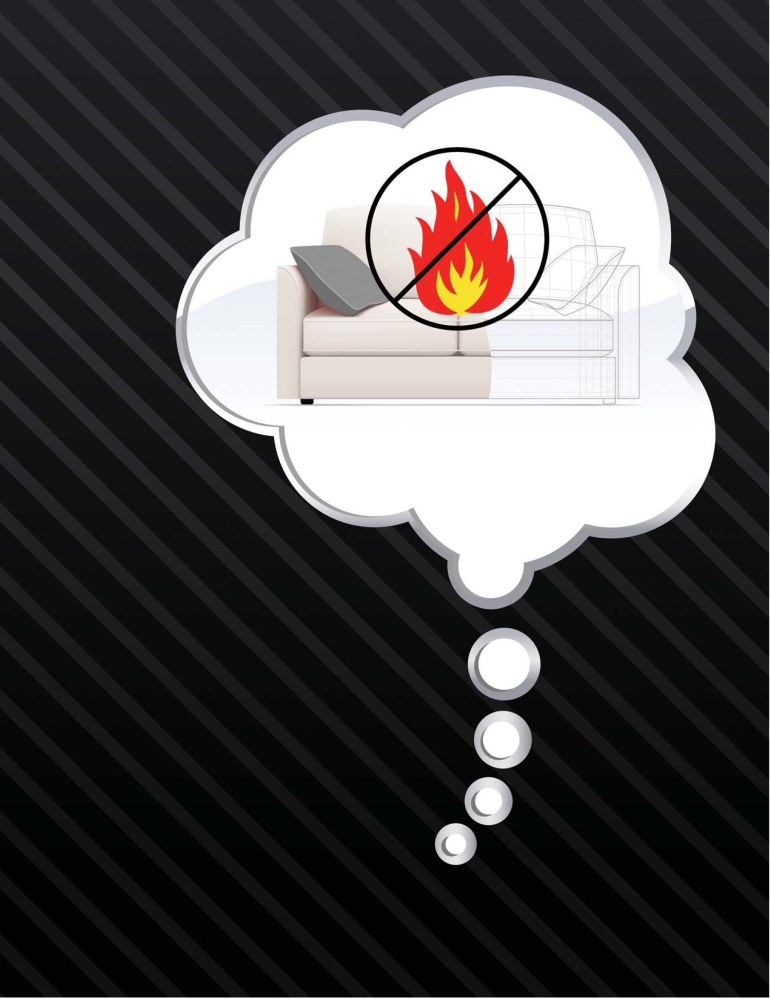
Although house dust is known to be a predominant source of exposure to PBDEs, it’s not yet clear which part of the dust these chemicals bind to. The dust pictured above contains pet hair (rust brown), pollen (yellow), plant fibers (green), dead skin cells (light to medium brown), dirt and minerals (orange), textile fibers (blue), and spider silk (pink).

## References

[b1-ehp0116-a00210] Kashiwagi T (2007). Flame retardant mechanism of the nanotubes-based nanocomposites.

[b2-ehp0116-a00210] Morgan AB, Wilkie CA (2007). Flame retardant polymer nanocomposites.

[b3-ehp0116-a00210] Nelson GL, Wilkie CA (2001). Fire and polymers: materials solutions for hazard prevention. ACS Symposium Series #797.

[b4-ehp0116-a00210] U.S. EPA (2005). Environmental profiles of chemical flame-retardant alternatives for low-density polyurethane foam, volume 2: chemical hazard reviews.

